# Supplementary material for: Should the Splenic Vein Be Preserved—Fate of Sinistral Portal Hypertension after Pancreatoduodenectomy with Vascular Re-Section for Pancreatic Cancer
Source: Cancers (Basel). 2022 Oct 4;14(19):4853. doi: 10.3390/cancers14194853 (PMC9564260; doi:10.3390/cancers14194853)
Supplement: Supplementary file 1 [file cancers-14-04853-s001.zip › cancers-1909343-supplementary-done.pdf]

cancers

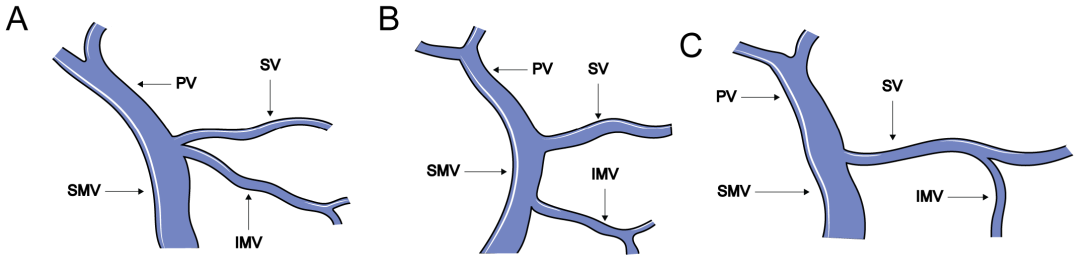

Figure S1. Inferior mesenteric vein insertion type: (**A**) type I, when the IMV drains into the PV/SMV junction, (**B**) type II, when the IMV drains into the SMV and (**C**) type III, when the IMV drains into the SV. IMV, inferior mesenteric vein; PV, portal vein; SMV, superior mesenteric vein; SV, splenic vein.

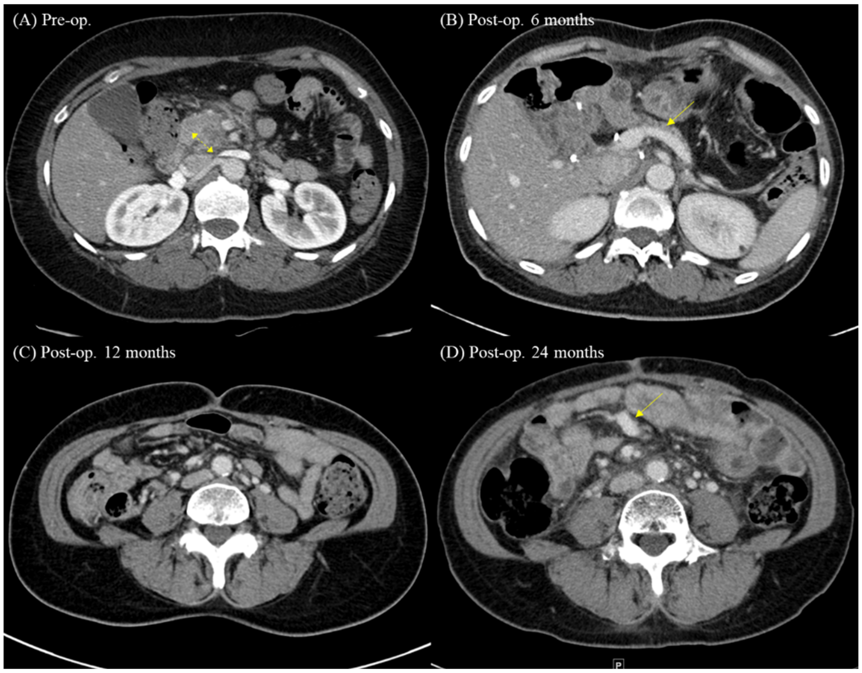

Figure S2. Representative images from the patient who underwent pancreatoduodenectomy of pancreatic cancer with tangential resection of the superior mesenteric vein, preserving splenic and inferior mesenteric veins. (A) A 55-year-old female patient shows an about 1.8-cm-sized pancreatic head cancer on preoperative CT (double-headed arrow). (B) On postoperative six-month CT, the splenic vein is preserved without luminal narrowing (arrow). The sum of the variceal scores was 0. (C) On postoperative 12-month CT, there was no new varix and the sum of the variceal scores remained 0. (D) On postoperative 24-month CT, an 8-mm dilated mesenteric vein is newly seen (arrow) without any other varices. The sum of the variceal scores increased to 2. There was no variceal bleeding event in this patient. Although R0 resection was achieved, the safety margin between the resected cancer and portal vein was as short as 250 μm. The final TNM stage was II (T1 N1). Disease-free survival and overall survival were 19 months and 35 months, respectively.

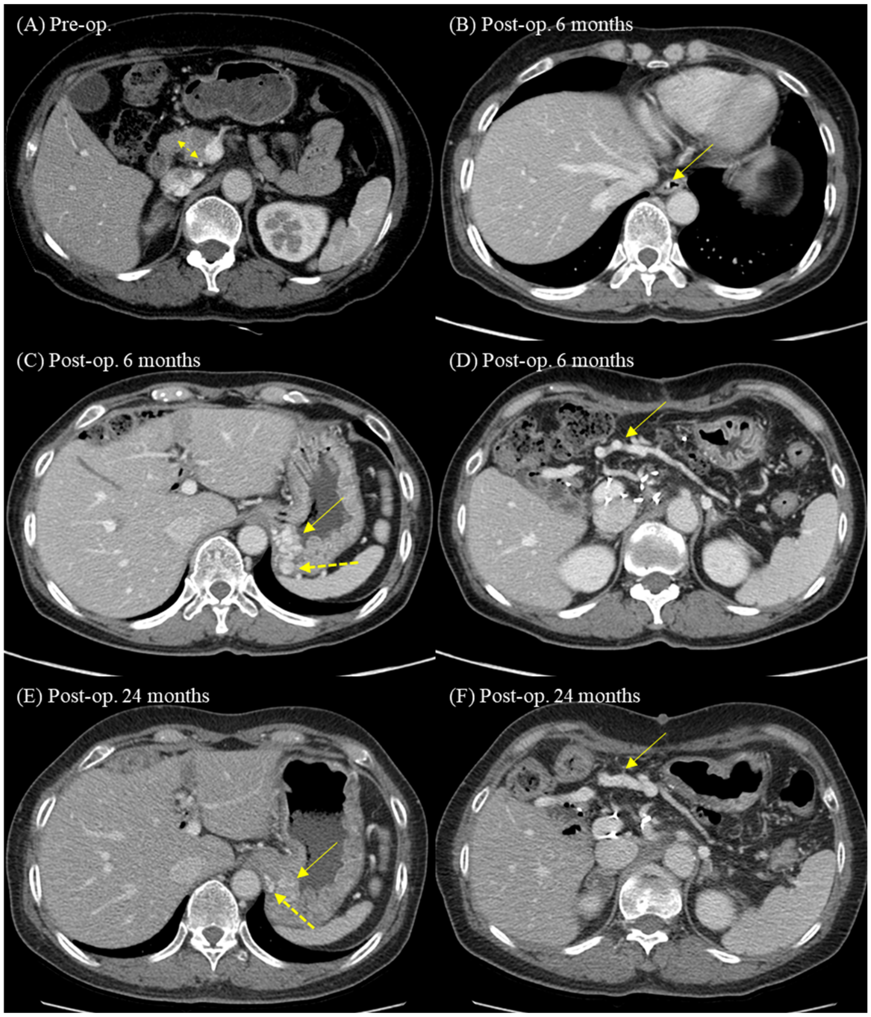

Figure S3. Representative images from the patient who underwent pancreatoduodenectomy of pancreatic cancer with segmental resection of the superior mesenteric vein and ligation of the inferior mesenteric and splenic veins. (A) A 57-year-old female patient shows an about 2.0-cm-sized pancreatic head cancer on preoperative CT (double-headed arrow). On postoperative 6-month CT, esophageal (variceal score: 1; arrow in [B]), gastric submucosal (variceal score: 3; arrow in [C]), gastric adventitial (variceal score: 2; dashed arrow in [C]), and mesenteric varices (variceal score: 3; arrow in [D]) newly appear. The sum of the variceal scores was 9, which remained the same at the postoperative 12-month CT (images not shown). (E) On the postoperative 24-month CT, gastric submucosal (arrow) and adventitial varices (dashed arrow) look less prominent. Scores of the gastric submucosal and adventitial varices changed to 0 and 2, respectively. Esophageal varix disappeared (image not shown) (F) Although mesenteric varices look slightly more prominent, the variceal score remained the same. The sum of variceal scores decreased to 5. There was no variceal bleeding event in this patient. R0 resection was successfully achieved, and the final TNM stage was I (T1 N0). Disease-free survival and overall survival were 57 months and 80 months, respectively.

Table S1. CT Criteria Used for Grading Varices.

Varices	Largest Diameter (mm)
Esophageal, paraesophageal, and gastric submucosal varices	
Grade	
0	< 2
1	2–2.9
2	3–6.9
3	≥ 7
4*	≥ 7
Gastric adventitial, splenic, mesenteric, retroperitoneal varices	
Grade	
0	< 3
1	3–4.9
2	5–9.9
3	≥ 10
4*	≥ 10

Note: If the number of dilated vessels on axial images is more than 4, the grade of varices increases one step higher. *Grade 4 was assigned when the number of grade 3 varices exceeded 4. *(Reference: Kim SH, Lee JM, Choi JY, et al. Changes of portosystemic collaterals and splenic volume on CT after liver transplantation and factors influencing those changes. AJR Am J Roentgenol 2008;191:W8-W16. doi:10.2214/AJR.07.2990).*
